# Multi-informant measurement of social anxiety symptoms in youth with social anxiety with and without autism

**DOI:** 10.3389/fpsyt.2025.1524088

**Published:** 2025-09-01

**Authors:** Marika C. Coffman, Ligia Antezana, Casper Brown, Amber Brown-Venegas, John A. Richey

**Affiliations:** ^1^ Department of Psychology, Virginia Tech, Blacksburg, VA, United States; ^2^ Duke University Center for Autism and Brain Development, Duke University, Durham, NC, United States; ^3^ Department of Psychiatry and Behavioral Sciences, Duke University, Durham, NC, United States; ^4^ Department of Psychiatry, University of Pittsburgh, Pittsburgh, PA, United States; ^5^ School of Social Work, University of Washington, Seattle, WA, United States

**Keywords:** parent-child agreement, autism spectrum disorder, social anxiety disorder, internalizing behavior, externalizing behavior

## Abstract

**Introduction:**

Discrepancies between caregiver and youth report of social anxiety symptoms persist, yet measuring social anxiety symptoms, particularly among autistic youths, is critical clinically to inform intervention planning and make correct diagnostic decisions.

**Methods:**

Accordingly, we sought to evaluate caregiver–adolescent agreement on measures of social anxiety across three diagnostic groups: (1) autistic, socially anxious adolescents (n=20), socially anxious, non-autistic adolescents (n=20), and a comparison group of non-autistic, non-socially anxious adolescents (n=20). Caregivers and adolescents completed the Anxiety Disorders Interview Schedule (ADIS), Social Anxiety module, and caregivers completed a battery of questionnaires to measure adolescent behavioral functioning in terms of adaptive, externalizing, and autism-related behaviors.

**Results:**

Compared with adolescents, caregivers generally indicated greater observed behavioral interference (e.g., avoiding preferred activities) on the ADIS due to social anxiety symptoms (*F*(1, 56) = 8.48, *p* < 0.01). Moreover, caregivers in the autistic group reported the highest level of behavioral interference, followed by the social anxiety group, and then the comparison group. Autistic adolescents and their caregivers had the poorest agreement for social anxiety symptoms compared with the other two groups.

**Discussion:**

These results demonstrate the differential impact of autism on the perception of social anxiety symptoms for caregivers and autistic adolescents. These results have implications for measuring social anxiety symptoms in autistic adolescents for research and clinical purposes as well as for intervention planning in this population.

## Introduction

Autistic adolescents experience high rates of co-occurring psychiatric disorders (1). Co-occurring anxiety disorders are common in autistic individuals (2), with higher levels of anxiety found in autistic compared with non-autistic youth (3). Discrepancies in reports of affective symptoms, symptom severity, and functional consequences between caregivers and youth is well studied (4–7). Internalizing symptoms show poorer agreement between caregivers and children, perhaps owing to the fact that symptoms are not as outwardly visible and children may not discuss internal states with others (8). This is further complicated by the clinical presentation of psychiatric disorders. Autism and anxiety may present with seemingly similar symptoms, but likely have manifold etiologic origins, which may be attributed differently by raters, serving to further reduce caregiver–child agreement of symptom types and severity. Studies have observed a mixed agreement between autistic youths and their caregivers on internalizing symptoms, with some research indicating that caregivers report higher internalizing symptoms (9), whereas others have found a higher adolescent report of symptoms (10).

Social anxiety symptomatology in the context of autism spectrum disorder may be particularly difficult to disentangle, due to overlapping social behaviors (11), despite high rates of co-occurrence. Social anxiety disorder (SAD) co-occurs with 42%–85% autistic individuals meeting diagnostic criteria for both conditions in their lifetime (12–14). Identification and diagnosis of SAD in autistic adolescents are complicated by a number of factors, however, including potential diagnostic overshadowing of similar behaviors across disorders (e.g., social withdrawal; 11), and differences in symptom reporting. Out of all anxiety disorder diagnoses, the potential for diagnostic overshadowing, or misconstruing social anxiety symptoms as autism behaviors or vice versa, is most likely to occur with social anxiety disorder (15). Accurate diagnosis is critical to inform appropriate interventions (16).

Only two studies have examined caregiver–youth agreement of social anxiety symptoms in a sample of autistic youths (17, 18). Burrows and colleagues (2018) found higher social anxiety symptoms reported in autistic youths. Schiltz and colleagues (2021) sought to determine the factor structure of a commonly used self- and other-report measure of social anxiety symptoms in autistic youths, with poor agreement between caregivers and youths on item-level reporting on the measure, and greater reporting of social anxiety symptoms by caregivers. These studies highlight the need for greater understanding of the differences in caregiver–youth reporting of social anxiety symptoms, particularly in autistic youths. These studies have not included a non-autistic comparison sample to determine whether differences in reporting are due to diagnostic overshadowing of autism-related behaviors.

Multiple factors are thought to influence agreement among raters for social anxiety symptoms, including age, sex assigned at birth, and cognitive ability (19–21). Increased age in general is associated with improved agreement between reporters (20), although for social anxiety specifically this is mixed, including both better (22) and worse agreement associated with age (23). Assigned sex at birth has also been associated with differences in reporting social anxiety symptoms, with better caregiver–child agreement among boys compared with girls (19, 23). Better cognitive ability has been associated with better caregiver–child agreement on anxiety symptoms in a sample of autistic children (24). Thus, it is important to examine demographic variables such as age, sex assigned at birth, and cognitive ability of the child when considering caregiver–youth agreement on anxiety symptoms.

Certain functional aspects of youth behavior, such as adaptive and externalizing behavior, have also demonstrated associations with social anxiety symptoms. Social anxiety can interfere with one’s adaptive behavior, including one’s ability to communicate effectively, complete daily living tasks, and socialize (17, 25). Burrows and colleagues (2018) found that higher agreement on social anxiety between caregivers and their autistic child was associated with better adaptive behaviors and autism characteristics, whereas in non-autistic children, higher agreement on social anxiety symptoms was associated with low adaptive behavior (17). Thus, there may be a differential relationship between the degree of agreement of social anxiety symptoms with adaptive behaviors in autistic compared with non-autistic children. Furthermore, externalizing symptoms tend to cause more overt distress and disturbance to family life and are more easily observed by caregivers. For example, externalizing symptoms may directly impact and change a caregiver’s plans (e.g., needing to end a grocery shopping trip early due to an outburst) but may serve a function for the child of avoiding an undesirable stimulus. Adaptive and externalizing behaviors may impact caregiver–youth agreement on social anxiety symptoms and may vary according to whether an adolescent has a co-occurring autism diagnosis and are important to consider when conceptualizing discrepant reports of caregiver and youth ratings of symptoms.

Current research in rater agreement emphasizes the need to better understand the cause of rater differences (26). While it has been appreciated for some time now that certain factors may enhance discrepancy in caregiver–adolescent reports of anxiety (particularly social anxiety) symptoms, and that these factors may be particularly pronounced in autistic populations, no study to date has examined these patterns in adolescents with social anxiety both with and without a diagnosis of autism. Adolescence in particular is an ideal time to examine social anxiety symptoms, as it increases in prevalence during this time period (27). We utilized a three-group sample to disentangle the impact of co-occurring social-behavior differences associated with autism, with (1) autistic, socially anxious adolescents (herein defined as “AUT+SAD”), (2) socially anxious, non-autistic adolescents (SAD), and (3) non-autistic, not socially anxious adolescents (herein defined as the comparison group, or "CG"). Accordingly, the present study aimed to replicate and extend prior work in examining group differences in caregiver-and-youth report of interference and clinical severity of social anxiety symptoms, and correlates of caregiver–youth agreement/disagreement. Based on previous work examining social anxiety in autistic and non-autistic samples, we predicted that compared with the comparison and SAD groups, the AUT+SAD group would have the highest caregiver ratings of social anxiety, although autistic youth themselves would report less social anxiety symptoms than the SAD group. Furthermore, consistent with previous work (19–21), we predicted that caregiver–youth agreement would be related to demographics (i.e., age, assigned sex at birth, IQ), adaptive behavior, externalizing behaviors, and autism characteristics.

## Methods

### Participants

Adolescents (N=60) ages 12 to 17 were enrolled in this study for three groups: AUT+SAD (n=20), SAD (n=20), and a comparison group of non-autistic, non-socially anxious adolescents (n=20). Notably, there was a significant sex difference in participants (*p* = 0.04). More autistic male than autistic female participants enrolled in the study, and more female than male participants were in the SAD group. These results are consistent with known diagnostic sex differences (28). A significant difference in IQ was also observed (*F*(2, 56) = 4.74, *p* = 0.01), such that the comparison group had significantly higher IQ scores compared with the AUT+SAD (*p* < 0.01), but not the SAD group (*p* = 0.22).

Demographic information for all participants is depicted in **Table 1**. Participants were recruited through mailing lists, flyers, and recruitment databases maintained through the Virginia Tech Psychology Department.

**Table 1:** Participant demographics.

	Total (N=60)	CG (n=20)	SAD (n=20)	Autistic (n=20)	*P-*value
Measure					
Age *Mean* (SD)	*14.83* (1.88)	*14.64* (1.92)	*14.89* (1.75)	*14.96* (2.03)	0.85
IQ *Mean* (SD)	*109.97* (13.73)	*116.40* (12.56)	*109.50* (12.16)	*103.68* (14.03)	0.01^*^
Sex					0.04^*^
Assigned Male at birth	31	11	6	14	
Assigned Female at birth	29	9	14	6	
Race/ethnicity					0.40
White	48	17	14	17	
Black	1	0	0	1	
Multiracial	6	3	3	0	
Asian	3	0	2	1	
Hispanic	2	0	1	1	

Means and standard deviations for age and IQ by group and ratios for sex assigned at birth and race/ethnicity are displayed above. Significance values are denoted such that *p<.05. IQ was measured with the WASI-II 2 subtest standard scores.

### Procedures

Interested families completed an eligibility phone screen, which included questions about their child’s behaviors related to autism and social anxiety, with the caregiver. Contingent on meeting inclusion criteria, participants completed two visits for the study. During the first visit, verbal and written caregiver consent and adolescent assent were obtained in accordance with the Virginia Tech IRB protocol (IRB #17-327). The first visit was an additional eligibility screening, consisting of caregivers and teens in all groups completing the Anxiety Disorders Interview Schedule, Fifth Edition (ADIS-5, 29) Social Phobia Module and the Wechsler Abbreviated Scale of Intelligence, Second Edition (WASI-II; 30). The ADIS-5 Social Phobia Module was completed with a center-research reliable graduate student. Autistic adolescents completed the Autism Diagnostic Observation Schedule, Second Edition (ADOS-2; 31) with a research reliable clinician. Participants also completed an fMRI during their second visit, although this work is focused on the behavioral characterization of the participants. Adolescents received $30 compensation for each study visit, for a total of $60 for study participation.

Participants were included in the study if they met the following criteria: aged 12-17, IQ of 80 or above, normal or corrected to normal vision, capable of making an informed decision (e.g., completing the fMRI). The AUT+SAD and SAD groups must have met criteria for a diagnosis of social anxiety as determined by meeting diagnostic criteria for SAD on the ADIS-5 SAD module by *either* the adolescent or the caregiver. The AUT+SAD group had an ADOS-2 score above clinical cutoff (i.e., at least “Autism Spectrum Disorder”).

Three participants were excluded from the sample. One autistic adolescent did not meet diagnostic criteria for SAD. One participant in the comparison group exhibited symptoms of anxiety in the fMRI experimental task and was thus excluded from this analysis out of an abundance of caution. One participant recruited in the autistic group completed diagnostic testing through a children’s developmental clinic and met diagnostic criteria for social anxiety based on parental report, meeting inclusion criteria for the experimental portion of the study. However, adolescent report was not collected, and thus their data are excluded from the present analyses.

### Measures

#### The anxiety and related disorders interview schedule for DSM-5

The ADIS-5, Child and Parent Versions (29) are semi-structured interviews for assessing general psychopathology in adolescents ages 12 to 17 years. The ADIS-5 has well-established reliability and validity (32). In the current study, only the SAD module was used. This module asks about several specific situations in which social anxiety symptoms may be present and asks the interviewee to rate the level of fear and avoidance associated with each situation. Caregivers and adolescents then provided the overall Interference score, which was a summative rating on how much social anxiety symptoms get in the way of the youth engaging in the things they like to do because of social anxiety. This rating was given on a scale of 0 = not at all/never to 8 = very severe. In addition to caregiver- and adolescent-reported Interference, clinicians assigned their own ratings of Severity via the Clinical Severity Rating of social anxiety. This summative score was informed by integrating information from both parent and youth report, as well as all available clinical observations. Severity ratings were similarly given on a scale of 0 = not at all to 8 = very severe.

#### Child Behavior Checklist

The Child Behavior Checklist (CBCL) (33) is a commonly used measure to identify and classify problem behavior in children. Caregivers completed the school aged version of the measure. The form consists of 120 statements regarding the child’s behavior, with ratings from 0 = not true, 1 = sometimes/somewhat true, and 2 = very true/often true. In accordance with guidelines from the manual, raw scores from the Externalizing scale were used as a dependent variable for our polynomial regression analysis.

#### Vineland Adaptive Behavior Scales, Third Edition

The Vineland-3 (34) is a measure used to assess individuals’ communication, social, and daily living skills across the lifespan. This study measured Adaptive Behavior using the Adaptive Behavior Composite (ABC) Standard Score.

#### Social Responsiveness Scale, Second Edition

The Social Responsiveness Scale, Second Edition (SRS-2) (35) is a well-validated measure of autism characteristics across settings. This measure detects subtle differences between autistic and nonautistic individuals. The SRS-2 consists of 65 items scored 1 = not at all true, 2 = sometimes true, 3 = often true, and 4 = almost always true. Scores are converted to subscales, awareness, cognition, communication, motor, and restricted and repetitive behaviors, as well as the total score. Raw scores were used in analyses as a measure of autism-related behavior, in alignment with recommended research practices (36).

#### Wechsler Abbreviated Scale of Intelligence, Second Edition

The WASI-II (37) is a structured measure of intelligence for individuals ages 6 to 90. This study used the 2-subtest Full Scale IQ Composite Score, using the scores from the Vocabulary and Matrix Reasoning subscales.

### Data analytic plan

Means and standard deviations are calculated for each group, and one-way ANOVAs are conducted to examine any group differences in age or IQ. Chi-square tests are used to examine differences in sex assigned at birth and race/ethnicity amongst groups.

In order to characterize the groups and test the first aim, related to group differences on caregiver and adolescent ADIS-5 Social Anxiety scores, a two-prong approach is utilized. First, 2 (Informant: Caregiver, Adolescent) × 3 (Group: AUT+SAD, SAD, CG) repeated-measures ANOVAs with Tukey *post-hoc* tests were conducted to examine (1) informant-rated Interference of social anxiety symptoms and (2) clinician-rated Severity of social anxiety symptoms. Effect sizes for Tukey’s *post-hoc* tests were reported with partial eta-squared (*η_p_^2^*) which ranges from 0 to 1, with values closer to 1 indicating a stronger effect of the factor. *η_p_^2^* between 0.01 and 0.06 is considered a small effect, *η_p_^2^* between 0.06 and 0.14, is considered a medium effect, and *η_p_^2^* > 0.14 is considered a large effect. Differences between adolescent and caregiver scores are visualized with group level data patterns.

Polynomial regressions are used for follow-up correlate analyses, which is appropriate to evaluate caregiver–adolescent agreement (38). In order to test the second aim, that demographics (age, sex assigned at birth, IQ), adaptive behavior, externalizing behavior, and autism characteristics are correlated with caregiver–adolescent agreement of social anxiety, a polynomial regression approach is used (38). This approach allows for testing an interaction between caregiver and adolescent reports and whether interactions associate with correlates of interest. Significant interaction terms indicate whether agreement/disagreement of caregiver- and adolescent-reported social anxiety relate to differences in the correlates of interest. Furthermore, it allows testing of this relationship above and beyond group, informant’s reports, quadratic terms of informant report, and group effects on informant report. Of note, quadratic effects are included to evaluate whether potential interaction terms are better explained by a nonlinear relationship. The following variables are included in the polynomial regressions: group linear and quadratic main effects of informant, two-way interactions (including group-by-informant interactions, caregiver-by-adolescent interactions), and three-way interactions (i.e., group-by-caregiver-by-adolescent interactions). These variables are consistent with previous research and variable selections in polynomial approaches in caregiver–child agreement analysis (17, 38). Interactions were probed using examination of scatter plots.

## Results

### Group differences in caregiver and adolescent ratings of social anxiety symptoms

#### Interference of social anxiety

**Figure 1:**
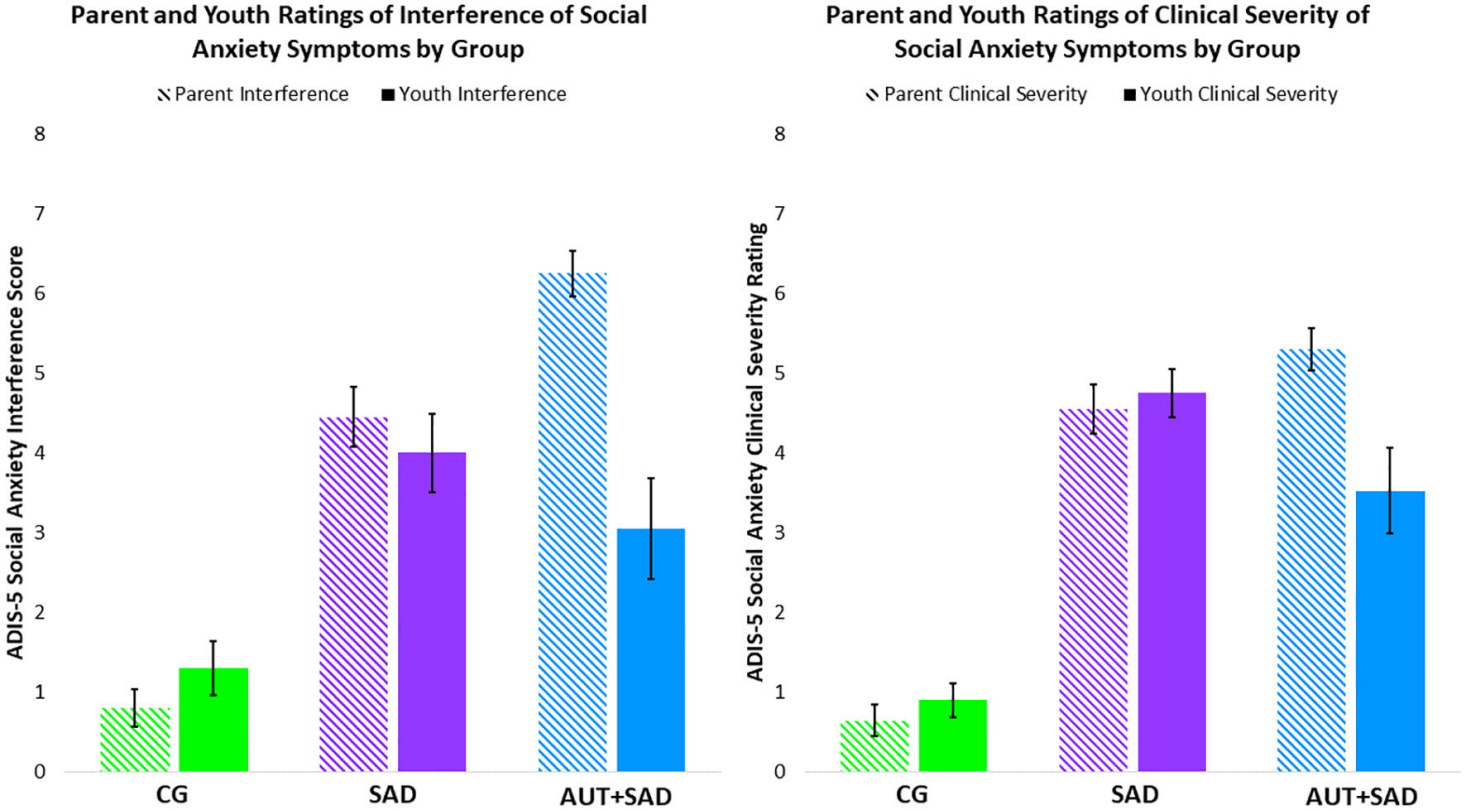
Bar graphs display caregiver and adolescent ADIS-5 Social Anxiety Interference and Severity Scores by group. The comparison group is depicted in green, the SAD group is depicted in purple, and the AUT+SAD group is depicted in blue. Caregiver ratings are in a lighter shading, whereas adolescent ratings are in the darker solid shading.

As expected, there was a significant main effect of Group for Interference of social anxiety symptoms, *F*(2, 56) = 49.07, *p* < 0.001, *η_p_^2^* = 0.64 (**Figure 1**). *Post-hoc* Tukey’s tests revealed that the comparison group had lower Interference scores than the autistic (Cohen’s *d* = 2.10) and SAD groups (Cohen’s *d* = 1.94; *p*s <.001), and the autistic and SAD groups did not differ from one another (*p* = .61). There was a main effect of Informant, *F*(1, 56) = 8.48, *p* < 0.01, *η_p_^2^* = 0.13, such that caregivers generally indicated greater interference of social anxiety symptoms than adolescents. A Group-by-Informant interaction was observed, *F*(2, 56) = 9.38, *p* < 0.001, *η_p_^2^* = 0.25. Follow-up one-way ANOVAs examining differences in caregiver and adolescent Interference are depicted in **Table 2**. For caregivers, all groups were significantly different from each other (*p*s <.001), such that caregivers in the autistic group reported the highest level of Interference, followed by the SAD group, and then the comparison group (AUT vs. SAD Cohen’s *d* = 1.21; AUT vs. the comparison group’s Cohen’s *d* = 4.62; SAD vs. the comparison group’s Cohen’s *d* = 2.61). For adolescents, the autistic (Cohen’s *d* =0.78) and SAD (Cohen’s *d* = 1.43) groups reported significantly more Interference than the comparison group (*p*s < 0.001), whereas the SAD group reported significantly more Interference than the autistic group (Cohen’s *d* = 0.38, *p* <.05).

**Table 2:** Group comparison of social anxiety and behavioral ratings.

Measure	CG (n=20)	SAD (n=20)	Autistic (n=20)	*P*-value
Caregiver and adolescent ratings of social anxiety				
Adolescent social anxiety interference *Mean* (SD)	*1.30* (1.53)	*4.00* (2.18)	*3.05* (2.768)	*0.001*
Range	0-5	0-8	0-8	
Caregiver social anxiety interference *M* (SD)	*.80* (1.06)	*4.45* (1.67)	*6.25* (1.29)	*<.001*
Range	0-4	1-7	4-8	
Adolescent social anxiety severity *Mean* (SD)	*.90* (.97)	*4.75* (1.37)	*3.53* (2.34)	*<.001*
Range	0-3	1-6	0-8	
Caregiver social anxiety severity *Mean* (SD)	*.65* (.88)	*4.55* (1.36)	*5.30* (1.17)	*<.001*
Range	0-3	1-6	3-8	
Caregiver ratings of adolescent behaviors				
Adaptive behavior *Mean* (SD)	*112.00* (10.13)	*102.89* (11.04)	*75.95* (8.42)	*<.001*
Range	84-124	85-121	58-91	
Externalizing behavior *Mean* (SD)	*3.20* (5.43)	*6.10* (8.06)	*14.10* (7.35)	*<.001*
Range	0-23	0-29	5-34	
Autism characteristics *Mean* (SD)	*19.70* (14.69)	*46.25* (26.12)	*104.15* (27.00)	*<.001*
Range	1-56	14-97	45-153	

Means, standard deviations, and ranges for ADIS-5 Social Anxiety Interference, ADIS-5 Social Anxiety Severity, Vineland-3 Adaptive Behavior Composite, CBCL Externalizing, and SRS-2 Autism Characteristics are displayed above by group.

#### Severity of social anxiety

As expected, there was a significant main effect of Group for Severity of social anxiety symptoms, *F*(2, 56) = 65.66, *p* < 0.001, *η_p_^2^* = 0.70. *Post-hoc* Tukey’s tests revealed that the comparison group had significantly lower scores than the autistic (Cohen’s *d* = 2.56), and SAD groups (Cohen’s *d* = 3.30, *p*s <.001), and the autistic and SAD groups did not differ from one another (*p* = 0.78). There was a main effect of Informant, *F*(1, 56) = 5.43, *p* < 0.05, *η_p_^2^* = 0.07, such that caregivers generally indicated greater Severity of social anxiety symptoms than adolescents. A Group-by-Informant interaction was observed, *F*(2, 56) = 10.17, *p* < 0.001, *η_p_^2^* = 0.27. Follow-up one-way ANOVAs with *post-hoc* Tukey’s test examining differences in Severity indicated that the autistic (Cohen’s *d* = 4.49) and SAD (Cohen’s *d* = 3.40) groups had significantly more caregiver-reported Severity than the comparison group (*p*s < 0.001), whereas the autistic and SAD groups did not differ in caregiver report (*p* >.11). For adolescent report of Severity, the autistic (Cohen’s *d* = 1.47) and SAD (Cohen’s *d* = 3.24) groups reported significantly more Severity than comparison group’ adolescents (*p*s < 0.001).

### Correlates of caregiver–adolescent agreement/disagreement

Results of the polynomial regression approach used to examine the relationship between caregiver–adolescent agreement and correlates of interest, which included demographics, adaptive behavior scores, externalizing behavior scores, and autism characteristics are documented in **Table 3**.

**Table 3:** Polynomial regression results with ADIS-5 Interference.

	Age		Sex assigned at birth		IQ		Adaptive behavior		Externalizing behavior		Autism Characteristics	
Predictor	*β*	*R^2^/ΔR^2^*	*β*	*Nagelkerke R^2^/ΔR^2^*	*β*	*R^2^/ΔR^2^*	*β*	*R^2^/ΔR^2^*	*β*	*R^2^/ΔR^2^*	*β*	*R^2^/ΔR^2^*
Step 1		.01/–		.16/–		.15^*^/–		.71^***^/–		.31^***^/–		.69^***^/–
Autistic group (dummy code)	−1.12		−9.54		**−**.73		−.92		.58		.99	
SAD group (dummy code)	−1.43^^^		−14.94^^^		.26		−.30		.42		−.14	
Step 2		.24^*^/.23^**^		.21/.05		.21^^^/.06		.73^***^/.02		.43^***^/.12^*^		.73^***^/.04
Adolescent social anxiety interference	1.04^^^		.72		.53		−.50		−.30		−.04	
Caregiver social anxiety interference	−.67		1.22		.40		−.84		.72		.05	
Adolescent social anxiety interference^2^	−.70		−.17^^^		−.48		.49		.43		−.08	
Caregiver social anxiety interference^2^	−.14		−.22		−.12		−.73		.37		.00	
Step 3		.27/.03		.41^^^/.20		.23/.02		.75^***^/.02		.44^**^/.01		.75^***^/.02
Autistic group × adolescent social anxiety interference	−.18		.37		.37		−.01		−.21		.17	
SAD group × adolescent social anxiety interference	.95		2.97^*^		−.19		−.08		−.67		.28	
Autistic group × caregiver social anxiety interference	1.97		1.47		.03		1.61		−.91		.001	
SAD group × caregiver social anxiety interference	1.80		3.04		−.67		1.49^		−.80		.49	
Caregiver social anxiety interference × adolescent social anxiety interference	.69		−.61		−.52		4.11^*^		−2.22		−.48	
Step 4		.29/.02		.41^^^/.00		.23/.00		.77^***^/.02		.45^**^/.01		.75^***^/.00
Autistic group × caregiver social anxiety interference × adolescent social anxiety interference	−.42		.63		.01		−3.72^^^		1.91		.16	
SAD group × caregiver social anxiety interference × adolescent social anxiety interference	−1.29		.10		.37		−3.17^*^		1.92		.07	

Regression results for associations between ADIS-5 Interference of Social Anxiety symptoms and demographics (age, sex assigned at birth, IQ), Vineland-3 Adaptive Behavior, CBCL Externalizing Behavior, and SRS-2 autism characteristics. Significance values are denoted such that ^^^p<.10, ^*^p<.05, ^**^p<.01, ^***^p<.001 and bolded values denote significance at the variable level.

#### Demographics

There was a main effect of adolescent-reported interference of social anxiety symptoms in the SAD group on sex assigned at birth (*p*<.05), such that participants assigned female at birth rated greater interference of social anxiety symptoms than participants assigned male at birth. No other significant associations were observed in age, sex assigned at birth, or IQ (*p*s > .05).

#### Adaptive behavior

**Figure 2:**
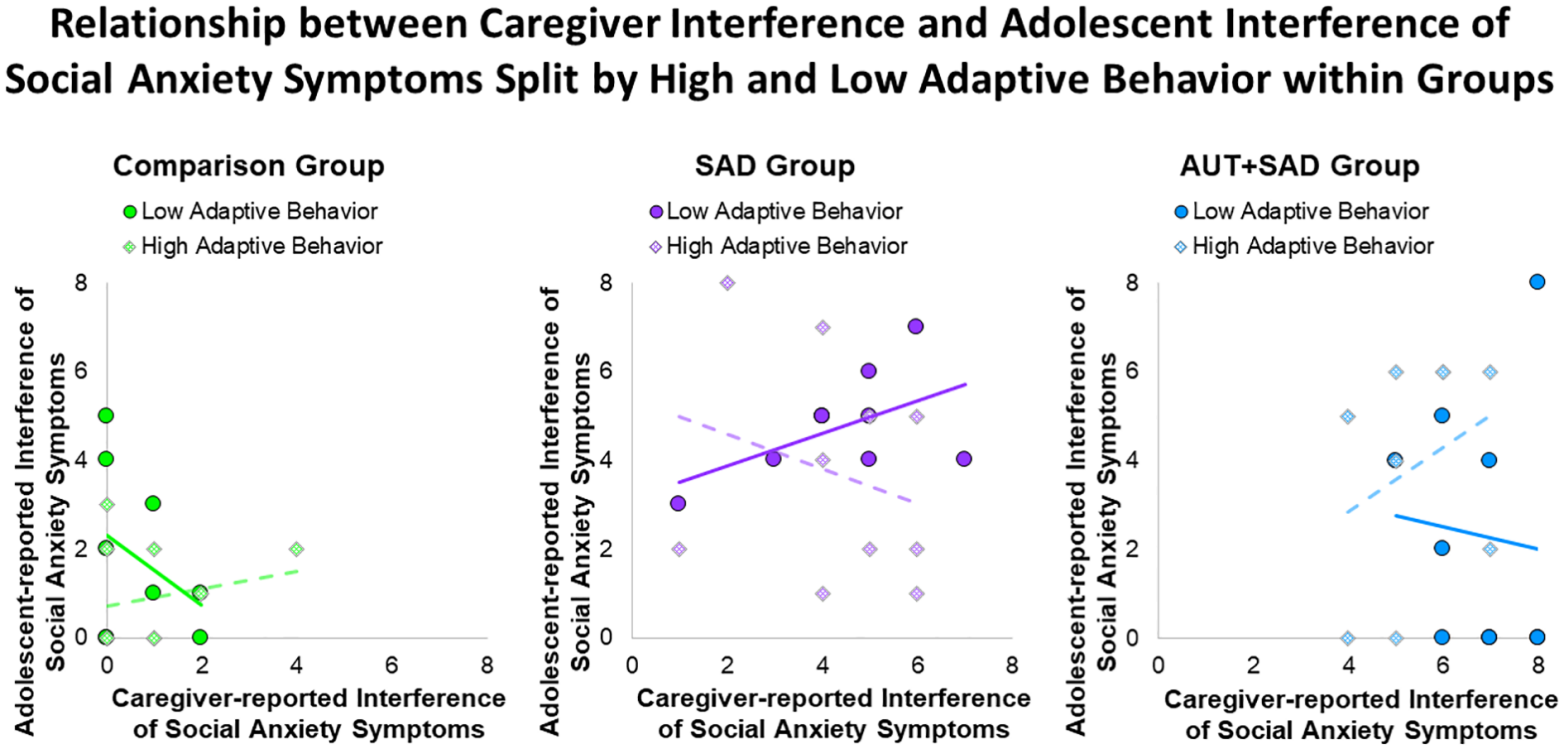
Scatter plots display interactions between Vineland-3 Adaptive Behavior and caregiver- and adolescent-reported ADIS-5 Social Anxiety Interference by group.

Adaptive behavior was associated with an interaction between informants, such that greater caregiver-adolescent agreement of interference was related to better adaptive behavior (*p* < 0.05). Additionally, a three-way interaction emerged for SAD group status by informants (*p* < 0.05). Exploratory examination of the role of adaptive behavior was probed by using a mean split by group (**Table 2**) with scatter plots (**Figure 2**). Examination of the plot within the SAD group showed that caregiver–adolescent agreement of social anxiety interference was related to low adaptive behavior, whereas caregiver–adolescent disagreement was related to high adaptive behavior (*p* < 0.05).

#### Externalizing behavior

Externalizing behavior was not associated with main effects of informant, interaction between informants, or interactions between groups and informants of social anxiety interference (*p*s > .39).

#### Autism characteristics

Autism characteristics were not associated with main effects of informant, interaction between informants, or interactions between groups and informants of social anxiety interference (*p*s > 0.20).

### Correlates of caregiver–adolescent agreement/disagreement of severity of social anxiety

#### Demographics

Age, sex, and IQ were not associated with main effects of informant, interaction between informants, or interactions between groups and informants for severity of social anxiety (*p*s > 0.05, **Table 4**).

**Table 4:** Polynomial regression results on associations between ADIS-5 Severity of Social Anxiety symptoms and demographics.

	Age		Sex assigned at birth		IQ		Adaptive behavior		Externalizing behavior		Autism characteristics	
Predictor	*β*	*R^2^/ΔR^2^*	*β*	*Nagelkerke R^2^/ΔR^2^*	*β*	*R^2^/ΔR^2^*	*β*	*R^2^/ΔR^2^*	*β*	*R^2^/ΔR^2^*	*β*	*R^2^/ΔR^2^*
Step 1		.01/–		.17^*^/–		.15^*^/–		.71^***^/–		.31^***^/–		.70^***^/–
Autistic group (dummy code)	−.95		−10.05		−1.14		−2.06^**^		.59		.48	
SAD group (dummy code)	−1.47		−1.13		1.61		−1.79		−.37		−2.54^*^	
Step 2		.21^*^/.20^*^		.22/.05		.20^^^/.050		.71^***^/.00		.39^***^/.08		.78^***^/.08^**^
Adolescent social anxiety severity	1.58^*^		−.81		.01		−.40		.26		.29	
Caregiver social anxiety severity	−.01		−.24		−.71		.21		.47		.22	
Adolescent social anxiety severity^2^	−.66		.13		−1.16		.96^^^		−.46		−1.21^**^	
Caregiver social anxiety severity^2^	−.76		−.26		−.28		−.11		.69		−.30	
Step 3		.30^^^/.09		.30/.08		.23/.03		.77^***^/.06^^^		.44^**^/.05		.80^***^/.02
Autistic group × adolescent social anxiety severity	.40		1.40		1.31		.77		.15		−.29	
SAD group × adolescent social anxiety severity	−.08		−.47		−1.18		1.38		.49		2.78^*^	
Autistic group × caregiver social anxiety severity	1.34		3.49		1.51		1.29		−.72		.44	
SAD group × caregiver social anxiety severity	1.38		2.12		−1.55		2.25		−.58		2.80	
Caregiver social anxiety severity × adolescent social anxiety severity	−2.58		−.01		4.13		−.69		−2.37		.02	
Step 4		.31/.01		.31/.01		.25/.02		.77^***^/.00		.44^**^/.00		.81^***^/.01
Autistic group × caregiver social Anxiety severity × adolescent social anxiety severity	1.64		.08		−4.02		−.90		1.60		1.04	
SAD group × caregiver social anxiety Severity × adolescent social anxiety severity	2.07		.65		–.66		–2.12		1.78		–2.03	

Regression results for associations between ADIS-5 Severity of Social Anxiety symptoms and demographics (age, sex assigned at birth, WASI-II IQ), Vineland-3 Adaptive Behavior, CBCL Externalizing Behavior, and SRS-2 behaviors associated with autism. Significance values are denoted such that ^^^p<.10, ^*^p<.05, ^**^p<.01, ^***^p<.001 and bolded values denote significance at the variable level.

#### Adaptive behavior

There were no significant main effects of informant, interaction between informants, or three-way interactions between group and informants of severity of social anxiety with adaptive behavior (*p*s > 0.05).

#### Externalizing behavior

Externalizing behavior was not associated with main effects of informant, interaction between informants, or interactions between groups and informants for severity of social anxiety (*p*s > 0.55).

#### Autism characteristics

There was an interaction between adolescent report of severity of social anxiety and SAD group status on SRS-2 scores, such that within the SAD group, those with greater severity of social anxiety based on adolescent report also had higher levels of autism characteristics (*p* < 0.05). No other significant associations were observed (*p*s > 0.20).

## Discussion

The present study used a semi-structured clinical interview of social anxiety symptoms in a sample of autistic adolescents with co-occurring social anxiety disorder, adolescents with social anxiety disorder, and a comparison group of non-anxious, non-autistic adolescents. We examined caregiver and adolescent ratings of interference and severity of social anxiety symptoms, caregiver–adolescent agreement, and correlates of caregiver–adolescent agreement of social anxiety. The autistic group demonstrated the greatest discrepancies in caregiver- and adolescent-reported scores of interference of social anxiety symptoms, as compared with socially anxious and non-socially anxious, non-autistic adolescents, with autistic adolescents reporting less interference than caregivers. A similar pattern emerged for clinician-reported social anxiety severity. Regarding correlates of caregiver–adolescent agreement/disagreement of social anxiety symptoms, adaptive behavior was associated with informant-rated interference of social anxiety symptoms, and not clinician-rated severity. This work provides potentially important insights into caregiver–adolescent agreement, particularly with understanding social anxiety in autistic adolescents. The greater disparities in agreement between caregivers and their autistic youths indicate a need to further probe social anxiety symptoms in the context of clinical work with these adolescents, and to potentially include multiple reporters for both research and clinical applications.

We found that caregivers of autistic youth reported the highest interference of social anxiety symptoms, followed by caregivers of youth with social anxiety disorder, and then the comparison group youth. Conversely, socially anxious youth reported the highest interference of social anxiety symptoms, followed by autistic youth and then the comparison youth. When examining caregiver–youth agreement of interference of social anxiety, the autistic group on average had the greatest discrepancies in agreement as compared with youth with social anxiety disorder and the comparison group youth. When examining clinician-rated severity of social anxiety symptoms, there was no significant difference between the autistic and SAD adolescents. The *Classifying Observations Necessitates Theory, Epistemology, and Testing (CONTEXT)* model posits that reporter discrepancies are meaningful and may be attributable to the context or situation in which the individual is reporting (39). When viewing our results in light of the CONTEXT model, it is important to consider that each reporter is observing social anxiety symptoms in a distinct setting and that all reports contain validity. Clinicians may serve as a buffer in parsing important information related to social anxiety vs. other social behaviors.

Correlates of caregiver–youth agreement/disagreement of interference of social anxiety symptoms within the socially anxious group demonstrated that greater caregiver–youth *disagreement* of interference was associated with better adaptive functioning, whereas in the autistic group, greater caregiver–youth *agreement* of interference was related to higher adaptive functioning. This work extends prior findings of Burrows and colleagues (2018), who examined parent–child agreement of social anxiety symptoms in autistic children, and found that better parent–child agreement of anxiety was associated with better adaptive behavior and autism characteristics. Our addition of the socially anxious, non-autistic group highlights a potentially unique role of adaptive behavior in social anxiety disorder with and without co-occurring autism. One potential reason for this difference is that, when adaptive behaviors are poor, caregivers and their autistic youths may not agree on causes of difficulties; conversely, in socially anxious, non-autistic youth, these may mask their symptoms of social anxiety.

Results from the current study indicated that when research-reliable clinicians were involved in evaluating both autism and social anxiety symptoms in an adolescent, the relationships observed between adaptive behaviors relative to social anxiety symptoms were altered. One possible explanation for this attenuation is that clinicians may be more focused on parsing out which social symptoms were attributable to social anxiety versus autism. Understanding the specific symptoms and attributing them correctly likely has important implications for interventions, as treating social anxiety symptoms will involve exposure therapy to a feared stimulus, which may be counterproductive if symptoms are social skill related (and thereby causing undue distress to the adolescent). Differences in caregiver and youth ratings between socially anxious and autistic groups were not present for severity of social anxiety symptoms, a rating given by a trained clinician based on an informant report. Despite no differences in caregiver and youth severity ratings between socially anxious and autistic groups, the autistic group had the highest discrepancies in caregiver–youth agreement of severity compared with both socially anxious and the comparison group.

Higher caregiver–adolescent agreement on social anxiety symptoms in adolescents may be related to clearer social behavioral expectations at that age (22). Anxious teens in our sample agreed with caregivers, which may be consistent with both groups understanding typical social norms and being aware of not meeting those expectations. In autism, it is possible that they are unaware of expectations or may not be interested in engaging, whereas caregivers observe a lack of meeting these behavioral expectations. For teens without autism and social anxiety, they may be hyper aware of their social performance in school or extracurricular environments in which their caregivers are likely not present.

Finally, we examined whether correlates previously associated with caregiver–child agreement, including demographics (age, sex assigned at birth, IQ), and externalizing behaviors, were observed in our sample. No relationship was found between caregiver–youth agreement/disagreement of severity and correlates of interests. This null finding may be due to a number of reasons and deserves further attention in future research. For example, the relatively narrow age range (12-17) may have limited possible age effects, and the number of comparisons to detect these nuanced associations may have been difficult to detect with the relatively limited sample size among the three group designs.

Results from the present study should also be viewed in terms of study limitations. Most notably, the sample was recruited to examine social anxiety in an autistic and non-autistic sample compared with peers without either psychiatric condition. Accordingly, the sample is potentially skewed such that the autistic sample has more social anxiety than is typical, with some other studies indicating 42%–85% of autistic individuals also meeting criteria for social anxiety disorder in their lifetime (12–14). The non-autistic sample was similarly recruited for both the presence and absence of social anxiety symptoms, leading to less range and variability in their social anxiety symptoms than is present in the general population. Only social anxiety was assessed via a clinical interview, which may result in non-diagnosis of other internalizing and externalizing disorders in the sample. Because the ADOS was only completed within the autistic group, it is possible that adolescents in the SAD and comparison groups had undiagnosed autism, although this was mitigated by the use of the phone screen and clinicians with experience working with autistic adolescents. The sample size is also relatively small by group, with only 20 adolescents per group. Moreover, it is important to be cognizant of the generalizability of the sample at large, but especially the autistic sample included here, as it was composed of autistic teens who are speaking and have and IQ≥80. It will be important for future studies to expand this work to adapt assessments for autistic individuals with less speaking abilities and the full range of IQ (e.g., using text or alternative communication to assess social anxiety) and also develop and validate observational measures of social anxiety to allow ability for multimodal assessment of SAD and, further, aid in the detection and intervention of co-occurring SAD for autistic teens across the spectrum.

It will also be important for future studies to further disentangle the driving factors of caregiver adolescent dis/agreement on social anxiety, particularly in autistic youth. This should include whether there are certain situations that are weighted more heavily by parents compared with their adolescents as being particularly informative for their perceptions of social anxiety symptoms. For example, are adolescents more attuned to public speaking and dating, whereas caregivers may find avoidance of eating in public or peer relationships as more salient? This is further compounded by heterogeneity in affective displays of fear (e.g., alexithymia, possible reduced facial expressions), behavioral manifestations of avoidance (e.g., elopement, stimming, differences in physical proximity), and behavioral features of present in autism (e.g., differences in verbal and nonverbal communication).

Despite these limitations, the current study offers several potentially important findings for caregiver–youth agreement across clinical disorders. We found that in socially anxious adolescents, caregivers tend to report greater interference of social anxiety symptoms and that this is heightened in autistic adolescents. However, this relationship was attenuated by clinician judgment. Attribution of social behaviors is critical for appropriate intervention planning. Future directions on this topic include further evaluation of the differences between reporters. Thus, including multiple viewpoints on child social anxiety symptoms provides important information for the context in which the symptoms are observed, consistent with the CONTEXT model (39). Examining caregiver, youth, and clinician report within the CONTEXT approach in the future may have an impact on therapeutic approaches for adolescents with social anxiety with and without autism.

## Data Availability

The raw data supporting the conclusions of this article will be made available by the authors, without undue reservation.
